# Pathogenic and Protective Roles of Neutrophils in *Chlamydia trachomatis* Infection

**DOI:** 10.3390/pathogens14020112

**Published:** 2025-01-23

**Authors:** Zoe E. R. Wilton, Andzoa N. Jamus, Susan B. Core, Kathryn M. Frietze

**Affiliations:** Department of Molecular Genetics and Microbiology, University of New Mexico Health Sciences, Albuquerque, NM 87131, USA

**Keywords:** chlamydia, *Chlamydia trachomatis*, neutrophil

## Abstract

*Chlamydia trachomatis* (Ct) is an obligate intracellular pathogen that causes the most commonly diagnosed bacterial sexually transmitted infection (STI) and is a leading cause of preventable blindness globally. Ct infections can generate a strong pro-inflammatory immune response, leading to immune-mediated pathology in infected tissues. Neutrophils play an important role in mediating both pathology and protection during infection. Excessive neutrophil activation, migration, and survival are associated with host tissue damage during *Chlamydia* infections. In contrast, neutrophils also perform phagocytic killing of *Chlamydia* in the presence of IFN-γ and anti-*Chlamydia* antibodies. Neutrophil extracellular traps (NETs) and many neutrophil degranulation products have also demonstrated strong anti-*Chlamydia* functions. To counteract this neutrophil-mediated protection, *Chlamydia* has developed several evasion strategies. Various *Chlamydia* proteins can limit potentially protective neutrophil responses by directly targeting receptors present on the surface of neutrophils or neutrophil degranulation products. In this review, we provide a survey of current knowledge regarding the role of neutrophils in pathogenesis and protection, including the ways that *Chlamydia* circumvents neutrophil functions, and we propose critical areas for future research.

## 1. Introduction

*Chlamydia trachomatis* (Ct) is an obligate intracellular bacterium with a biphasic lifecycle that infects mucosal epithelial cells in humans, mainly at the urogenital and anorectal tracts and the eye. *Chlamydia* elementary bodies (EBs), which are the infectious and non-replicative form, bind to suitable host cells through interactions with host heparan sulfate proteoglycans and various host cell receptors [1,2,3]. Ct then utilizes a type III secretion system (T3SS) to inject effector proteins into the host cell cytosol, inducing cytoskeletal rearrangements and facilitating bacterial internalization as a membrane-bound inclusion [1]. In the inclusion, EBs differentiate into reticulate bodies (RBs), which are the replicating noninfectious form of the bacterium [1,2,3,4]. Once enough RBs have accumulated through replication, the RBs convert back into EBs [1,2,3,4,5]. The EBs can then exit the host cell through lysis or extrusion, enabling the release of *Chlamydia*-secreted proteins and the continuation of the infectious cycle [1]. 

There are three human biovars of Ct, which infect different mucosal sites throughout the body [1,6]. The genital biovar, consisting of serovars D-K, is sexually transmitted. In 2016, there were 127 million newly diagnosed cases of sexually transmitted Ct, which rose to 129 million new cases in 2020 [7,8,9]. Infection of the female reproductive tract (FRT) can lead to long-term sequelae including pelvic inflammatory disease (PID), ectopic pregnancy, and tubal factor infertility [1,10]. 

While Ct infections can be cleared using antibiotics, many individuals are still susceptible to reinfection [11,12,13,14]. In addition, around 77% of Ct-infected individuals are asymptomatic, which can delay STI testing and treatment [15]. For these reasons, the development of a vaccine is crucial to combat the continuing rise in *Chlamydia* infections. Extensive efforts in recent years to develop a vaccine against urogenital Ct infection culminated in two Phase 1 human clinical trials of a vaccine that targets an immunodominant antigen called the major outer membrane protein (MOMP) [16]. However, there remains no licensed vaccine for urogenital Ct infections.

Understanding the immune response to *Chlamydia* infection is important for developing vaccines. Animal models and natural history studies of *Chlamydia* infection have revealed interferon gamma (IFN-γ)-producing CD4+ T cells as correlates of protection against re-infection [13,17,18,19,20,21]. However, many types of immune cell play conflicting roles in both protection and immunopathology during Ct infection [14,22,23,24,25,26,27,28]. In this review, we focus on the contradictory role of neutrophils in contributing to protection against and the pathology of *Chlamydia,* as well as the mechanisms by which *Chlamydia* can modulate neutrophil functions. Finally, we propose areas for future research efforts.

## 2. Neutrophil Functions in Host Response to Pathogens

Neutrophils are the most abundant leukocytes in humans [29]. Neutrophils, a type of polymorphonuclear cell (PMN), are an essential part of the innate immune response and are frequently thought of as a “first line of defense” against pathogens [30]. When *Chlamydia* infections occur, circulating neutrophils are rapidly recruited to the *Chlamydia*-infected epithelium. Recruitment of circulating neutrophils is initiated by the release of various chemokines and cytokines, such as interleukin 8 (IL-8), from *Chlamydia*-infected epithelial cells [30,31,32]. The release of these cytokines promotes the activation of endothelial cells, which line blood vessels that circulating neutrophils travel through, leading to increased surface exposure of adhesion molecules present on endothelial cells, such as vascular cell adhesion molecule1 (VCAM-1/CD106) [33]. These adhesion molecules then bind to ligands present on circulating neutrophils to promote tethering and rolling along vascular and/or endothelial surfaces [30,33,34,35]. The luminal surface of the endothelium then expresses chemokines that can activate neutrophils to induce a conformational change, which allows previously circulating neutrophils to exit the bloodstream and gain entry into the site of *Chlamydia* infection [30]. The main mechanisms of neutrophil-mediated protection are phagocytosis, neutrophil extracellular trap (NET) release, and degranulation [30,36,37]. If excessive, these mechanisms of protection can also promote host tissue damage. 

### 2.1. Phagocytosis

Phagocytosis is a mechanism utilized by neutrophils to eliminate pathogenic microbes and remove debris to maintain tissue homeostasis [30,38,39]. Neutrophils engulf external particles or cells using their plasma membrane, which can occur via two different mechanisms: (1) the plasma membrane can form actin-based protrusions that surround the external material, or (2) the plasma membrane can fully wrap around the particle due to binding between neutrophil cell surface receptors and external particle ligands [30,40]. These particles are transported into the cell in vesicles called phagosomes [30]. The phagosome fuses with pre-formed granules that contain anti-microbial enzymes [30,31,41]. Nicotinamide adenine dinucleotide phosphate (NADPH) oxidase subunits are also translocated to the phagosome, enabling the assembly and generation of reactive oxygen species (ROS), which function to kill phagocytosed microorganisms [42]. This results in the formation of an autophagosome and the degradation of the internalized particles. 

Neutrophils have several ways to recognize potentially pathogenic microbes that should be phagocytosed, including recognition of immunoglobulin G and A (IgG and IgA, respectively) antibodies that have opsonized pathogens [40]. These antibodies interact with Fc receptors on the surface of neutrophils to promote internalization [30,38]. The presence of pro-inflammatory cytokines tumor necrosis factor-α (TNF-α) and interleukin 1β (IL-1β) can increase Fc-mediated phagocytosis by neutrophils [30,43,44]. Additionally, pathogens can also be opsonized with complement and interact with β2 integrins on neutrophils to promote phagocytosis [38,45]. 

### 2.2. NETosis

NETs are constructed from chromatin that is mainly obtained from neutrophil nuclear DNA that has relaxed and formed a complex with various antimicrobial proteins and proteases [37,46]. Some mitochondrial DNA can also be found in NETs [37,47]. NETosis, the release of NETs into the extracellular space, creates sites of highly concentrated antimicrobial proteins that promote the immobilization and destruction of microbes [30,37,46]. The presence of NETs can also modify the innate immune system by promoting the production of pro-inflammatory cytokines [39]. The formation of NETs can be triggered by various stimuli, such as antibody opsonization of a pathogen and the presence of cytokines [37,48]. 

NETs can be formed in two different ways. The first is rapid formation, which is independent of ROS and occurs within a few minutes of stimulation [37,49,50]. This can result in vital/non-lytic NETosis, when NETs are released from living cells via secretion of both chromatin and the contents of granules [30,37,50]. Interestingly, neutrophils that perform vital NETosis can still perform other anti-microbial functions such as phagocytosis and migration [37,48,51]. This rapid production of NETs is thought to rapidly immobilize microorganisms so that they can be killed by other mechanisms of neutrophil-mediated protection [51]. The second form of NETosis is much slower and occurs over a period of several hours [37]. This slower formation of NETs utilizes ROS to release proteins in the azurophilic granule of neutrophils, allowing the newly released neutrophil elastase (NE) to cleave F-actin, dissociating the cytoskeleton and immobilizing the neutrophils [31,48]. Proteins released from the azurophil granule, such as myeloperoxidase (MPO), peptidyl arginine deaminase 4 (PAD4), and NE, then enter the nucleus to cause nuclear decondensation through a variety of methods [31,37,48]. After the nuclear envelope disassembles, decondensed nuclear chromatin enters into the cytoplasm where it interacts with both cytoplasmic and granule contents [37]. The plasma membrane of the neutrophil then becomes permeable, releasing NETs into the extracellular space and causing neutrophil cell death [37]. This slower form of NETosis has been well studied [37,49]. 

### 2.3. Degranulation

Neutrophil degranulation is the release of anti-microbial proteins and peptides, and it is frequently dependent on the presence of ROS [32,42]. Degranulation occurs through the mobilization and fusion of pre-formed granules, which contain antimicrobial products, to the phagosome or the plasma membrane. Mobilization and fusion with the plasma membrane allow the granule products to be released outside of the cell [30,31,47,52]. The contents of these pre-formed granules have strong anti-microbial functions, including the ability to directly kill microorganisms and/or degrade the extracellular matrix (ECM) to allow increased leukocyte infiltration at the site of infection [32,53,54,55,56]. 

Neutrophil granules are typically classified into four main types: primary, secondary, tertiary, and secretory [47]. Primary/azurophilic granules contain many pro-inflammatory and anti-microbial products, such as defensins, NE, and MPO. Primary granules require a strong stimulus to trigger release into the extracellular space, and many of these granule products can also damage host cells [30,32]. Secondary granules and tertiary granules also contain anti-microbial products that are released into the extracellular space after stimulation. Secondary granules contain lysozyme, pre-cathelicidins, and lactoferrin [30,32]. Tertiary granules contain the proteolytic enzyme matrix metalloproteinase 9 (MMP9) and cathepsin [30,32]. During the standard degradation response, primary and secondary granules are mainly directed to the phagosome instead of outside the cell, to minimize damage to host tissue [30,52]. However, when granule contents are released outside the cell, tertiary granules are typically released first, followed by secondary granules, and then primary granules [32,53]. Secretory granules products, such as Fc and complement receptors, are continuously released to replenish receptors on the cell surface of the neutrophils [32,52]. 

## 3. Contributions of Neutrophils to *Chlamydia*-Associated Pathology

While neutrophils are well-known mediators of protection during pathogenic infections, neutrophil-mediated mechanisms of protection can induce host cell damage. In fact, neutrophils are typically associated with the development of immunopathology during *Chlamydia* infection. 

Neutrophil depletion studies conducted in animal models strongly support this association, as several models have shown that neutrophil depletion decreases *Chlamydia*-associated pathology despite having no impact on bacterial burden. Lee et al. found that neutropenic mice that were vaginally infected with *Chlamydia muridarum* (Cm), a well-established mouse model of infection that mimics Ct ascension and pathology in humans, displayed decreased levels of hydrosalpinx compared with neutrophil-competent mice, despite having a similar bacterial burden [57]. Hydrosalpinx is a fluid blockage in the upper FRT, which serves as a marker for infertility and tissue fibrosis and is associated with chronic *Chlamydia* infections in mice [57]. It is important to note that neutropenia was induced using anti-Gr-1 RB6-8C5 monoclonal antibody treatment, which recognizes both Ly6G and Ly6C. This means that certain subpopulations of macrophages, monocytes, lymphocytes, and dendritic cells might have also been affected by this depletion [57]. Lijek et al. found that neutrophil-depleted mice that were trans-cervically infected with Ct serovar D (CtsvD) had significantly decreased upper FRT pathology compared with neutrophil-competent mice [58]. Similar to the study by Lee et al., neutrophil depletion was not shown to have a significant impact on bacterial burden in this model [58]. Neutrophil depletion was achieved using either anti-Gr-1 or anti-Ly6G, a more neutrophil-specific monoclonal antibody treatment. Both of these depletion methods resulted in a similar decrease in immunopathology, indicating that neutrophils are the main mediator of pathology in mouse models of genital *Chlamydia* infections [58]. Neutrophils can also contribute to *Chlamydia*-associated pathology during ocular infections [59]. Lacy et al. found that depletion of neutrophils resulted in decreased ocular pathology in a guinea pig model of *Chlamydia caviae* inclusion conjunctivitis [59]. Similar to the genital infection models, neutrophil depletion seemed not to impact bacterial burden but impacted the development of *Chlamydia*-associated pathology [59]. These models of neutrophil depletion strongly indicate that neutrophils are major mediators of *Chlamydia*-associated pathology during infection.

### 3.1. Increased Neutrophil Recruitment, Activation, and Survival Can Promote Chlamydia-Associated Pathology

NK cells and neutrophils are the first immune cell types recruited to the site of *Chlamydia* infection [21]. Neutrophils are recruited to the site of infection by epithelial cells that secrete pro-inflammatory cytokines and chemokines during *Chlamydia* infection, such as IL-8 (also called chemokine (C-X-C motif) ligand 8 (CXCL8)), IL-6, CXCL1, CXCL2, and CXCL5 [60,61,62,63]. In addition to cytokines and chemokines, *Chlamydia*-infected epithelial cells can secrete granulocyte macrophage colony-stimulating factor (GM-CSF), which promotes neutrophil migration and survival [62]. 

Prolonged neutrophil influx, activation, and survival during *Chlamydia* infection may increase the risk of *Chlamydia*-associated pathology. A study carried out by Lee et al. found that decreased neutrophil recruitment to the site of infection decreased *Chlamydia*-associated pathology in the upper FRT [63]. This was determined by infecting mice that were genetic knockouts (KOs) for the chemokine (C-X-C motif) receptor 2 (CXCR2) or their wild-type (WT) counterparts with Cm [62,63]. CXCR2 is a major chemokine receptor present on the surface of neutrophils that interacts with CXCL1-CXCL3 and CXCL5-8 to mediate recruitment and activation [63]. It is important to note that knocking out CXCR2 could impact other cell types, such as monocytes, that express this chemokine receptor [64]. However, the decrease in upper FRT pathology found in this mouse model is still likely caused by disrupted neutrophil recruitment. This was indicated by the CXCR2 KO mice having decreased neutrophils and neutrophil-secreted MMP-9 levels in their FRT despite having increased peripheral neutrophil levels [63].

Increased neutrophil activation during *Chlamydia* infection is also associated with an increased risk of *Chlamydia*-associated pathology [23]. In a study of women at increased risk for PID, Wiesenfeld et al. found a correlation between increased levels of neutrophil alpha defensins, a marker of neutrophil activation, in the vaginal tract and the development of endometritis after Ct infection [23,65].

Increased neutrophil survival might also promote *Chlamydia*-associated pathology. Prolonged neutrophil survival is associated with tissue damage during other pathogenic infections, as apoptosis is thought to be a critical mechanism of halting neutrophil activation in an anti-inflammatory manner [66,67,68]. *Chlamydia* infection can prolong neutrophil survival, and thus prolong neutrophil activity [69]. Frazer et al. showed that when partially purified human neutrophil cultures were incubated with CtsvD, there was a significant decrease in the percentage of apoptotic neutrophils compared with cultures incubated with cell media alone [23]. Altogether, these studies demonstrate the relationship between *Chlamydia*-associated pathology development and neutrophil recruitment, activation, and survival.

### 3.2. The Release of ROS by Neutrophils as a Contributor to Immunopathology

Mechanistically, neutrophils can contribute to *Chlamydia* pathology in many ways. One-way neutrophils can contribute to *Chlamydia*-associated pathology is through the secretion of ROS. ROS are produced by many different cell types in response to *Chlamydia* [70,71]. While ROS have bactericidal abilities that may provide protection, ROS release can also directly damage host tissue and promote inflammation by increasing neutrophil activation. ROS are known to promote neutrophil priming, phagocytosis, degranulation, and NET release, which can damage host tissue [42,72]. In addition, ROS can activate neutrophil degranulation products such as MMP-9 [73]. The relationship between ROS secretion by phagocytes and *Chlamydia*-associated pathology has been shown in mouse models of infection. p47^phox^ is a key component of phagocyte NADPH oxidase and is critical for priming ROS production. Ramsey et al. found that p47^phox^ KO mice had decreased hydrosalpinx formation compared with WT mice when intravaginally infected with Cm [74]. It is important to note that this model included knocking out ROS production for all phagocytic cells, not neutrophils specifically. 

### 3.3. Neutrophil Degranulation as a Contributor to Immunopathology

Neutrophil degranulation products, such as MMPs, can contribute to *Chlamydia*-associated pathology. MMPs are a family of enzymes involved in the degradation of ECM, the processing of chemokines and cytokines into their active form, and promoting the recruitment of leukocytes [75,76]. In healthy human women, MMPs play important roles in the menstrual cycle and reproductive cycle [77]. MMPs also play an important role in protection during pathogenic infections. During infections, MMPs are rapidly secreted, typically by neutrophil degranulation, and rapidly activated.

During *Chlamydia* infection, neutrophils are the primary producer of MMP-9 (also known as gelatinase B) [23,78]. This was indicated by a decrease in the total and active levels of MMP-9 during neutrophil depletion in Cm infected mice [57]. MMP-9 is strongly associated with the development of *Chlamydia*-associated pathology, such as scarring and fibrosis of the murine oviduct [23,76]. This was reported by Imtiaz et al., who found that MMP-9 KO mice had significantly decreased hydrosalpinx formation at 56 days post-infection (d.p.i.) with Cm compared with WT mice [76]. MMP-9 can contribute to *Chlamydia*-associated pathology in several ways. MMP-9 is a type IV collagenase that can degrade ECM components such as denatured collagens and native collagen type IV. These make up the basal lamina to which columnar epithelial cells in the uterus and fallopian tubes are attached [57,76]. Increased levels of MMP-9 may promote epithelial necrosis and sloughing in the FRT during *Chlamydia* infection, allowing dissemination of infection as infected epithelial cells are released [57,76,78]. Excessive ECM degradation has been linked to increased fibrosis during tissue repair, contributing to hydrosalpinx formation after *Chlamydia* infection [76,78]. MMP-9 can also regulate various chemokines and cytokines, such as CXCL1, CXCL5, CXCL7, CXCL8, TNF-α, IL-1β, and transforming growth factor β (TGF-β) [57,73,79,80]. This can promote a positive feedback loop of recruitment and activation of immune cells at the site of *Chlamydia* infection, thus increasing inflammation-related damage [57,76,79].

In addition to MMP-9, other MMPs may play a role in promoting *Chlamydia*-associated pathology. A study by Ault et al. found increased expression of active MMP-9 and MMP-2 when human fallopian tube tissue was cultured with Ct, compared with culturing in cell media alone [81]. Similar to MMP-9, MMP-2 is a type IV collagenase that can degrade ECM components when activated by neutrophils [82]. The association between MMPs and *Chlamydia*-associated pathology was supported by a mouse model of MMP inhibition. Imtiaz et al. found that there was a reduction in inflammation, ascension of infection, and severity of hydrosalpinx when mice were treated with a global inhibitor of MMPs, chemically modified tetracycline (CMT-3), compared with MMP-9 KO mice [75,76]. CMT-3 treatment significantly decreased both MMP-9 and MMP-2, implying that multiple MMPs might promote *Chlamydia*-associated pathology [75,76]. This could have been due the ECM degradation abilities of the MMPs and/or the ability of other MMPs to activate MMP-9 [73]. 

### 3.4. Neutrophil Phagocytosis as a Contributor to Immunopathology

Neutrophils are professional phagocytic cells that can uptake and kill various pathogens. Previous papers have reported that neutrophils can uptake *Chlamydia*, especially in the presence of *Chlamydia*-specific antibodies [83,84]. However, phagocytosis is not always associated with immune-mediated protection. Yu et al. found that increased antibody-mediated neutrophil phagocytosis of *Chlamydia* was not correlated with clinical outcomes of protection (spontaneous resolution or protection against reinfection) in human females [85]. In addition to not being associated with protection, neutrophil phagocytosis may be detrimental to the host. *Chlamydia* EBs can survive in neutrophils for several hours while maintaining their infectious capabilities [29,86]. Surviving *Chlamydia* can then escape neutrophil phagocytosis by promoting cell cytotoxicity [29,86]. This is detrimental as the *Chlamydia* EBs may escape phagocytosis as the neutrophils travel through previously uninfected areas of the FRT, promoting dissemination of infection [26,29,75]. Uptake by a phagocyte may also provide *Chlamydia* physical protection against other immune cell types [26]. In addition, cellular products released from neutrophil cell death could serve as damage-associated molecular patterns (DAMPs), which can stimulate the release of pro-inflammatory cytokines by macrophages, such as IL-6 and IL-8 [86]. As these cytokines can promote the recruitment of neutrophils, this could cause a positive feedback loop that encourages a chronic inflammatory environment and immunopathology [86].

### 3.5. NET Release as a Contributor to Immunopathology

**Figure 1:**
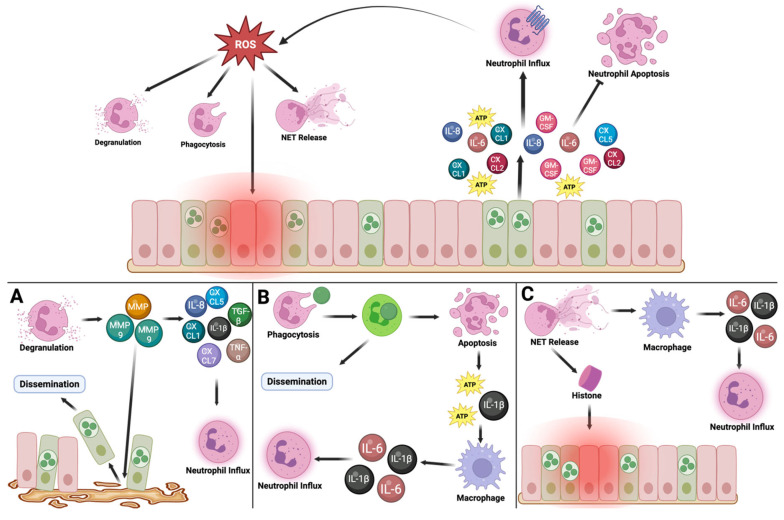
Schematic illustrating how *Chlamydia* infections can activate neutrophils, which can contribute to pathology and host tissue damage. Top: Epithelial cells are infected with *Chlamydia*, which causes the release of pro-inflammatory cytokines and chemokines such as IL-8, IL-6, GM-CSF, and CXCL1. These cytokines and chemokines promote the adhesion of circulating neutrophils to endothelial cells. The circulating neutrophils then undergo conformational change that allows them to exit the bloodstream and enter the site of *Chlamydia* infection. The cytokines also promote the activation and survival of present neutrophils. These activated neutrophils increase production of ROS, which can cause directly oxidative damage to host cells. ROS can also damage host cells by promoting neutrophil phagocytosis, degranulation, and NET release. (**A**) Neutrophil degranulation can damage host cells by releasing of MMPs, such as MMP-9, which can disrupt the basal lamina that the FRT epithelial cells are anchored to. This can cause the epithelial cells to disseminate, potentially allowing spread of the infection. In addition, degradation of the basal lamina by MMPs can promote fibrosis during tissue repair. MMPs are also able to regulate different chemokines and cytokines, which can increase neutrophil recruitment to the site of infection. (**B**) *Chlamydia* can survive in neutrophils for several hours; therefore, neutrophil phagocytosis can contribute to pathology by allowing *Chlamydia* to disseminate to other areas of the FRT by utilizing neutrophils as a method of transportation and protection against immune surveillance. Infected neutrophils can also die in response to infection, thus releasing DAMPs that promote macrophage activation and cytokine release. Several of these macrophage-secreted cytokines can promote neutrophil recruitment, thus triggering a positive feedback loop. (**C**) NET release can directly and indirectly damage host cells. NET release causes histones and anti-microbial products to be released into the extracellular space, where they can be cytotoxic to other cells and/or function as DAMPs to promote a pro-inflammatory environment. Macrophages also release pro-inflammatory cytokines in response to NETs, promoting neutrophil influx.

NET release is another way that neutrophils can contribute to host tissue damage during infection. NETs can both directly and indirectly damage host tissue. Many products released into the extracellular environment during NETosis, such as histones, can cause cell cytotoxicity at high concentrations [37,48,87]. NETosis products can also act as DAMPs, promoting chronic inflammatory environments by stimulating the production of pro-inflammatory cytokines by macrophages [37,88,89]. In addition, NET release can promote T-cell activation and differentiation into Th17 cells under some inflammatory conditions [89]. This can be detrimental during *Chlamydia* infection, as increased T-cell differentiation into Th17 cells is associated with non-protective and/or immunopathogenic response [24,28,90,91]. The association between Th17 cells and immunopathology might be caused by the production of IL-17 by Th17 cells. This could promote a positive feedback loop, as IL-17 stimulates neutrophil generation, maturation, and mobilization [24] (Figure 1).

## 4. Protective Roles of Neutrophils Against *Chlamydia* Infection

Despite the strong association between neutrophils and *Chlamydia*-associated pathology, several recent studies have indicated that neutrophils might play a role in protection in the presence of *Chlamydia*-specific antibodies [92]. 

There has been a long-standing desire to define the role of neutrophils during *Chlamydia* infections, with early papers from the 1990s using improved antibody technology to define this role. Barteneva et al. found that the depletion of granulocytes using monoclonal antibody RB6-8C5 resulted in an increased rate of infection and increased early bacterial burden in mice that received primary intravaginal challenges with Ct [93]. This is contradicted by more recent research demonstrating that neutrophils play an important protective role in infection only in the presence of *Chlamydia*-specific antibodies. Naglak et al. showed that mice depleted of neutrophils prior to a secondary Cm challenge had increased bacterial burden compared with neutrophil-competent mice [84]. Naglak et al. also reported that neutrophils were protective only during early secondary infection with *Chlamydia* [84]. This was based on results showing a delay in clearance and increased inclusion-forming units (IFUs) in T-cell-deficient mice that were depleted of neutrophils during early secondary infection compared with T-cell-deficient mice that were depleted of neutrophils later during infection [84]. While these studies established the importance of neutrophils when *Chlamydia*-specific antibodies are present, the exact mechanism of neutrophil-mediated protection is still unclear.

### 4.1. Neutrophil Degranulation as a Contributor to Immune-Mediated Protection

Neutrophil degranulation may play a role in immune-mediated protection during *Chlamydia* infection. Many components of neutrophil granules have anti-*Chlamydial* functions, such as lactoferrin. Lactoferrin is a protein that is released from mucosal epithelial cells and the secondary granules of neutrophils during infection [94]. Elevated levels of lactoferrin are found in the cervicovaginal fluid of women with Ct infections compared with noninfected women [95]. This increase in lactoferrin levels, potentially by neutrophil degranulation, could be beneficial; lactoferrin can prevent *Chlamydia* infection of host cells in vitro [96]. This is potentially due to lactoferrin interacting with host cell receptors, such as heparan sulfate receptors, that are utilized for Ct adhesion [1,94,97]. Increased lactoferrin levels could also be beneficial, as lactoferrin has anti-inflammatory properties [94]. Lactoferrin can downregulate inflammation by regulating free iron levels, thus limiting ROS production that could damage host tissue and promote the release of pro-inflammatory cytokines [94]. Indeed, a study by Sessa et al. found that the addition of lactoferrin decreased the production of pro-inflammatory cytokines IL-6 and IL-8 by Ct-infected host cells in vitro [96]. This study also indicated that lactoferrin could promote Ct clearance in vivo [96]. In a small study of pregnant women infected with Ct, Sessa et al. found that intravaginally administered bovine lactoferrin resulted in Ct clearance and decreased IL-6 levels in six out of the seven patients enrolled [96]. 

Additionally, other degranulation proteins are protective against *Chlamydia*, such as defensins and cathelicidins released from mucosal epithelial cells and the secondary granules of neutrophils [98]. One particular cathelicidin produced by human neutrophils is LL-37 (called CRAMP in mice/rats), which showed strong anti-*Chlamydia* activity in vitro [99]. LL-37 plays an important role in regulating innate immunity and can function as a natural broad-spectrum antibiotic that neutralizes lipopolysaccharides (LPS) present on Gram-negative bacteria [32,100]. 

### 4.2. Neutrophil Phagocytosis as a Contributor to Immune-Mediated Protection

Phagocytosis and phagocytic killing are thought to be possible mechanisms by which neutrophils can provide protection against *Chlamydia* [7,83,84]. Previous studies have found that Ct EBs pre-incubated with immune sera containing *Chlamydia*-specific antibodies were more readily phagocytosed by neutrophils, compared with Ct EBs that had been incubated with immune sera absent of *Chlamydia*-specific antibodies [83,101]. In previously infected humans, there was a positive correlation between *Chlamydia*-specific IgG antibody titers and neutrophil phagocytosis [85,101]. 

Naglak et al. found that neutrophils increased the phagocytic killing of *Chlamydia* EBs in the presence of both IFN-γ and *Chlamydia*-specific antibodies, compared with neutrophils that were stimulated with only cell media [84]. It is important to note that phagocytic killing by neutrophils was increased only in the presence of both IFN-γ and *Chlamydia*-specific antibodies, and there was no increase in phagocytic killing by neutrophils in the presence of *Chlamydia*-specific antibodies alone [84]. This is similar to other bacterial pathogens that display increases in neutrophil phagocytosis and killing in the presence of IFN-γ [102]. The exact mechanism of phagocytic killing in response to IFN-γ is currently unknown in the case of *Chlamydia*; thus, further exploration is needed. IFN-γ has been shown to increase neutrophil phagocytic killing of other bacteria through both oxidative and non-oxidative mechanisms [102]. The presence of IFN-γ might aid the phagocytic killing of *Chlamydia* by upregulating FcγRI on neutrophils to promote ROS production and phagocytosis of IgG-opsonized *Chlamydia* [103]. 

### 4.3. NET Release as a Contributor to Immune-Mediated Protection

**Figure 2:**
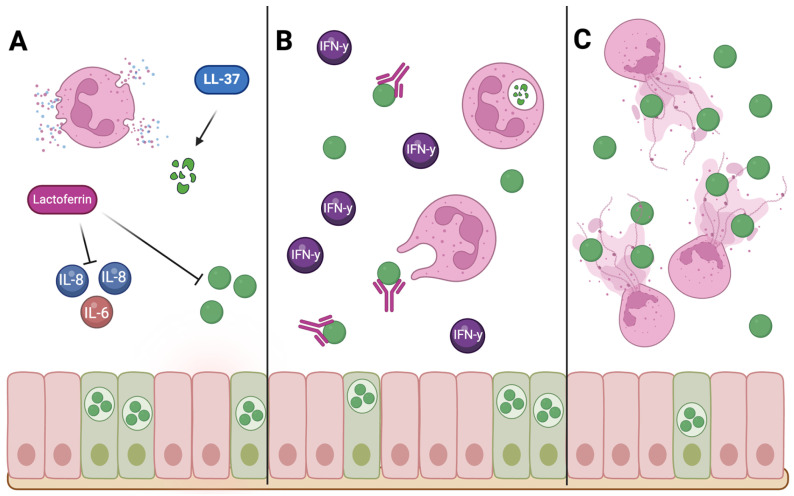
Schematic illustrating neutrophil-mediated mechanisms of protection against *Chlamydia*. Neutrophils can provide protection by three main mechanisms: degranulation, phagocytosis, and NET release. (**A**) During degranulation, neutrophils can release human cathelicidin LL-37, which has strong anti-*Chlamydia* activity. Lactoferrin is also released during degranulation, which can block entry of *Chlamydia* into host cells and decrease the secretion of pro-inflammatory cytokines during infection, potentially reducing tissue damage. (**B**) Neutrophils can also provide protection against *Chlamydia* infection by phagocytosing and killing the engulfed bacteria in the presence of IFN-γ and *Chlamydia*-specific antibodies. (**C**) NET release, which can directly kill and/or prevent the dissemination of pathogens, is another way that neutrophils might provide protection.

Neutrophils can also provide protection through the release of NETs. Rajeeve et al. reported rapid clearance of *Chlamydia* in the absence of *Chlamydial* protease-like activity factor (CPAF), a *Chlamydia*-secreted protein that inhibits NET release and degranulation [52,99,104]. The rapid clearance of these CPAF-deficient mutants indicates that neutrophils can provide protection, potentially through the release of NETs or degranulation products [104] (Figure 2).

## 5. Modulation of Neutrophils by *Chlamydia* Proteins

### 5.1. Chlamydial Protease-like Activity Factor (CPAF)

In order to prevent elimination by neutrophils, *Chlamydia* has several strategies to evade or manipulate neutrophil-mediated mechanisms of protection. The most well-defined of these strategies is the utilization of CPAF, which is a type II secretion system effector that is secreted into the cytoplasm of host cells and released when infected cells lyse [1]. CPAF is crucial for *Chlamydia*’s survival, as indicated by CPAF-deficient mutants being rapidly eliminated in both cell cultures and mouse models [104,105]. An immune response against CPAF, elicited by intranasal immunization with a recombinant CPAF protein adjuvanted with CpG deoxynucleotides (CpG), can reduce *Chlamydia* bacterial burden and pathology in mouse models, which further emphasizes the importance of CPAF in *Chlamydia* infections [88,106]. CPAF can play numerous roles during *Chlamydia* infection, including maintaining *Chlamydia* inclusion integrity in the host cell, promoting the survival of *Chlamydia*, helping fragment the host cell Golgi, and promoting host cell lysis [1]. CPAF also plays a role in disrupting host immune protection through downregulating antigen presentation molecules, downregulating transcription factors that promote host cell immunity, and manipulating the response of neutrophils [1,107,108,109]. 

Rajeeve et al. found that CPAF promotes extended survival of *Chlamydia* in PMNs and prevented neutrophil-mediated mechanisms of protection [104]. Rajeeve et al. found that neutrophils infected with CPAF-competent Ct displayed decreased expression of neutrophil activation marker CD11b, decreased production of ROS, and decreased NET release [104]. This decrease in neutrophil activity occurred even in the presence of phorbol myristate acetate (PMA), which can directly activate neutrophils and stimulate NET release. This was in contrast to neutrophils infected with a CPAF-deficient Ct mutant that readily became activated, produced ROS, and released NETs in response to stimulation, with or without the presence of PMA [104]. Rajeeve et al. also found that neutrophils infected with CPAF-competent Ct had decreased expression of CD35, a marker of neutrophil degranulation, compared with neutrophils infected with the CPAF-deficient Ct mutant [52,104]. The paper showed that CPAF cleaves the G-coupled receptor formyl peptide receptor 2 (FPR2) present on the neutrophil cell surface, which mediates activation and induction of antimicrobial responses [104]. FPR1, which has similar functions, was not shown to be affected [104]. Cleavage of FPR2 can disrupt calcium mobilization and downstream signaling pathways, such as phosphatidylinositol-3-OH kinase (PI3K) and MAP-kinase (MAPK) signaling, which promote production of ROS and pro-inflammatory cytokines [52,104]. This disruption to ROS production and calcium mobilization impedes neutrophil degranulation and NET release [52,104,110]. While Rajeeve et al. found that CPAF prevented neutrophil activation, ROS production, NET release, and degranulation, the presence of CPAF did not impact the capability of neutrophils to perform phagocytosis [104]. However, inhibition of FPR2 can reduce neutrophil phagocytosis of other pathogenic bacteria, such as *Escherichia coli*, even when FPR1 is still present [111]. So, there is the potential that CPAF could impact neutrophil phagocytosis to a small extent. Altogether, these results indicate that CPAF can directly manipulate neutrophils to support the survival of *Chlamydia*. 

Tang et al. found that CPAF can also degrade antimicrobial peptides, such as human cathelicidin LL-37, which are secreted mainly by epithelial cells and neutrophils in response to *Chlamydia* infections [99]. LL-37 is stored in the secondary granules of neutrophils and can eliminate microbes by inducing pore formation in bacterial membranes. LL-37 has strong anti-*Chlamydia* functions that can protect host cells from being infected; however, when the LL-37 peptide was pre-incubated with CPAF, there was no detectable protection against *Chlamydia* infection in vitro [99,112]. This reduction in LL-37 protection was due to CPAF degrading LL-37 [99]. CPAF can also degrade other peptides with anti-Chlamydial functions in vitro, although to a lesser extent than LL-37 [99]. The authors concluded that CPAF can preferentially degrade antimicrobial peptides that have strong anti-*Chlamydia* functions to enhance *Chlamydia*’s survival and spread in the extracellular environment, thus promoting ascension of infection [99].

### 5.2. Plasmid Gene Protein 3 (Pgp3)

Other *Chlamydia* effector proteins can also inhibit the protective functions of LL-37. Pgp3 is part of the cryptic plasmid of *Chlamydia* [113]. The cryptic plasmid is highly conserved and made up of eight open reading frames (ORFs) named pORFs 1-8 or Pgp 1-8, which contribute to virulence and pathology [113]. *Chlamydia* that is depleted of the cryptic plasmid or deficient in Pgp3 can still infect host cells but has limited ascension to the upper FRT and does not induce upper genital tract or ocular pathology [113,114]. Pgp3 is secreted from the inclusion into the host cytosol during the late stages of infection [113]. When host cells undergo lysis at the end of the infectious cycle, Pgp3 is released into the extracellular environment [113]. Once in the extracellular environment, Pgp3 interacts with LL-37 to form a stable complex (Pgp3/LL-37) that can reduce the anti-Chlamydial functions of LL-37 and modulate the immune response [113,114]. Hou et al. showed that the presence of LL-37 alone decreased CtsvD infection of HeLa cells in vitro. However, when LL-37 was preincubated with Pgp3, there was a dose-dependent increase in CtsvD infection [114]. Hou et al. also showed that interactions between Pgp3 and LL-37 could delay neutrophil recruitment to the site of infection by reducing LL-37’s ability to induce pro-inflammatory cytokine secretion in human vaginal epithelial cells [113]. This resulted in reduced neutrophil migration in the presence of Pgp3/LL-37 complexes compared with free LL-37 [113]. In addition to impacting the release of pro-inflammatory cytokines by vaginal epithelial cells, Pgp3/LL-37 complexes can also impact the release of pro-inflammatory cytokines by neutrophils. Pgp3/LL-37 complexes were shown to stimulate neutrophils to secrete increased levels of pro-inflammatory cytokines associated with *Chlamydia* pathogenesis, such as IL-8 and IL-6 [113]. The ability of Pgp3/LL-37 complexes to promote pro-inflammatory cytokine secretion by neutrophils while preventing the secretion of the same pro-inflammatory cytokines in vaginal epithelial cells might seem contradictory. However, the authors believe that this dual role might enhance *Chlamydia*’s survival by delaying inflammation and innate immune cell recruitment until the infection is better established [113]. After this, inflammation could be beneficial to promote spreading of *Chlamydia* by innate immune cells such as macrophages and neutrophils [29,75,113]. Due to the importance of Pgp3 in promoting *Chlamydia* pathology and manipulating the host immune response, Pgp3-deficient *Chlamydia* mutants are being investigated as a potential candidate for a live attenuated vaccine [115].

### 5.3. Chlamydia High-Temperature Requirement Protein A (cHtrA)

cHtrA is a *Chlamydia* effector protein that can inhibit the protective functions of LL-37 [112]. cHtrA is a serine protease that is secreted by *Chlamydia* into the host cytosol, where it can degrade ECM components, promote invasiveness, and function as a chaperone [112,116]. Dong et al. found that purified cHtrA protein could also cleave LL-37 in vitro, thus preventing its anti-Chlamydial function [112]. When LL-37 was present without cHtrA, there was a decrease in the number of HeLa cells infected with *Chlamydia* [112]. However, preincubation of LL-37 with HtrA resulted in a dose-dependent increase in *Chlamydia* infection in vitro [112]. As previously mentioned, the degradation of antimicrobial peptides by *Chlamydia*-secreted proteins could promote survival, spread, and ascension [112].

### 5.4. CT135

CT135 is a Ct inclusion membrane protein and virulence factor that is important for the development of *Chlamydia*-associated pathology in the upper FRT. Mice infected transcervically with CT135-deficient Ct had reduced uterine pathology and infertility, indicated by the number of embryos per mouse, compared with mice that were infected with CT135-competent Ct [86]. This difference in pathology could be accredited to the anti-neutrophil function of CT135, which promotes neutrophil cell death [86]. Yang et al. found that mouse bone marrow-derived neutrophils (BMDNs) that were infected with CT135-competent Ct had increased cytotoxicity compared with CT135-deficient Ct at the same multiplicity of infection (MOI), as indicated by lactate dehydrogenase (LDH) release [86]. Yang et al. showed that toll-like receptor 2 (TLR2)/MyD88 signaling, which promotes NLRP3/caspase1 inflammasome activation, was activated in response to CT135 and promoted neutrophil cell death [86]. This was supported by in vitro studies showing that BMDNs isolated from TLR2-/-, MyD88-/-, NLRP3-/-, and caspase1-/- mice had decreased levels of the pro-inflammatory and pro-apoptotic cytokines IL-1, IL-1β, and IL-6 compared with infected WT BMDNs when infected with CT135-competent CtsvD [86,117,118]. The authors of the paper postulated that CT135 might function as a pore protein, transporting fragments of the Ct lipoprotein omcA from the inclusion into the neutrophil cytosol, which could then be bound by TLR2 and activate the MyD88 signaling pathway [86]. The paper also indicated that DAMPs, such as extracellular ATP released by infected neutrophils and epithelial cells, promote the release of pro-inflammatory cytokines associated with *Chlamydia* pathology by macrophages [86]. Altogether, this indicates that CT135 can directly trigger neutrophil cell death, resulting in the release of DAMPs that promote a pro-inflammatory environment. 

**Figure 3:**
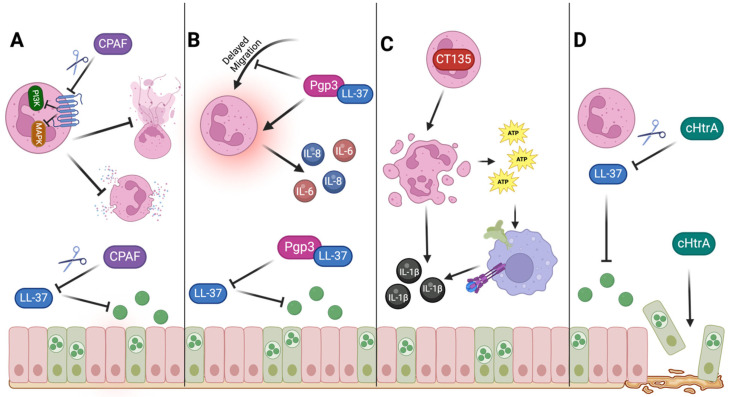
Schematic illustrating how *Chlamydia* can disrupt neutrophil-mediated protection. (**A**) CPAF can cleave the FPR2 receptor on the surface of neutrophils, which disrupts PI3K and MAPK signaling. The disruption of these signaling pathways can prevent neutrophils from degranulating or performing NET release. Another mechanism of anti-neutrophil activity by CPAF is the degradation of LL-37. This prevents LL-37 from being able to perform anti-*Chlamydia* functions, enhancing host cell infection. (**B**) Pgp3 can form a stable complex with LL-37, limiting the anti-*Chlamydia* functions of LL-37. The presence of Pgp3 can directly interfere with LL-37’s ability to prevent *Chlamydia* infection in host cells, and Pgp3/LL-37 complexes can delay neutrophil migration to the site of infection. Pgp3, free or complexed with LL-37, promotes the release of the pro-inflammatory cytokines IL-6 and IL-8 by neutrophils, which can contribute to the pro-inflammatory environment at the site of infection. (**C**) CT135 can destroy neutrophils that have taken up *Chlamydia* EBs. The presence of CT135 can trigger neutrophil cell death through TLR2/MyD88 signaling, which triggers the NLRP3 inflammasome. This results in the release of pro-inflammatory cytokines, such as IL-1β, and DAMPs, such as extracellular ATP. Macrophages can recognize the presence of extracellular ATP through the P2X7 receptor, which results in inflammasome activation and the release of pro-inflammatory cytokines, such as IL-1β. (**D**) cHtrA can cleave LL-37, thus preventing the anti-*Chlamydia* functions of LL-37. In addition, cHtrA can also degrade ECM components, which could promote *Chlamydia* spreading as infected cells are released and travel through the FRT.

These studies show that *Chlamydia* has developed several mechanisms to circumvent neutrophil-mediated protection. A deeper understanding of these various strategies could provide therapeutic targets to promote *Chlamydia* clearance by neutrophils and limit the contributions of neutrophils to *Chlamydia*-associated pathology. Additionally, several of these proteins have shown promise as a prophylactic *Chlamydia* vaccine (Figure 3).

## 6. Future Directions for Research and Implications for *Chlamydia* Vaccine Development

More research is needed to fully elucidate the role of neutrophils during *Chlamydia* infection. Currently, there is strong evidence that supports the role of neutrophils in promoting *Chlamydia*-associated pathology. However, even though neutrophils have been shown to be protective in the presence of *Chlamydia*-specific antibodies, there has been limited investigation into neutrophil-mediated protection during infection. A more in-depth examination of potential mechanisms of neutrophil-mediated protection is imperative. Fully understanding the contradictory role of neutrophils during *Chlamydia* infection would provide insight into how *Chlamydia* can modify or avoid potentially protective host immune responses. As many cell types play contradictory roles in protection and pathology during *Chlamydia* infection, the potentially protective role of neutrophils should not be dismissed just because of the association with *Chlamydia*-associated pathology.

A deeper examination of the relationship between neutrophils and *Chlamydia* could be beneficial for the development of a protective vaccine. This is emphasized by the existence of several promising *Chlamydia* vaccine candidates that target proteins known to disrupt neutrophil functions. Currently, there is a limited breadth of knowledge on how the presence of vaccine-elicited antibodies can impact the response of neutrophils to *Chlamydia* infection. It would be interesting to examine whether neutrophils are still associated with increased pathology in vaccinated mice, or whether the potentially shorter duration of infection limits the continuous activation of neutrophils that is associated with pathology. Additionally, it would be interesting to examine the impact of vaccine-elicited antibodies on neutrophil-mediated mechanisms of protection, as this might differ from the effects of antibodies acquired during natural infection, due to more specific targeting and increased antibody titers. 

## 7. Conclusions

Neutrophils play an important role in both *Chlamydia* protection and pathology. During primary infection when *Chlamydia*-specific antibodies are not present, the influx of neutrophils into the FRT stimulates the production of pro-inflammatory cytokines and tissue-damaging molecules that promote pathology. However, in the presence of *Chlamydia*-specific antibodies, neutrophils play an important role in protection. To prevent this, *Chlamydia* has developed several strategies to diminish or skew neutrophil-mediated mechanisms of protection. Currently, several *Chlamydia* vaccine candidates that target proteins known to affect neutrophil functions have shown promise in mouse models [88,106,115]. Understanding the potentially important role of neutrophils in antibody-mediated protection could inform strategies to design safe and efficacious vaccines and/or therapeutic targets against Ct.
